# Preparation and Characterization of Ferulic Acid Wheat Gluten Nanofiber Films with Excellent Antimicrobial Properties

**DOI:** 10.3390/foods12142778

**Published:** 2023-07-21

**Authors:** Chengming Jin, Huijuan Zhang, Feiyue Ren, Jing Wang, Sheng Yin

**Affiliations:** China-Canada Joint Laboratory of Food Nutrition and Health (Beijing), Key Laboratory of Special Food Supervision Technology for State Market Regulation, School of Food and Health, Beijing Technology and Business University (BTBU), Beijing 100048, China; 18811784478@163.com (C.J.); zhanghuijuan@th.btbu.edu.cn (H.Z.); 20200101@btbu.edu.cn (F.R.); wangjing@th.btbu.edu.cn (J.W.)

**Keywords:** wheat gluten (WG), ferulic acid, electrospinning, antimicrobial

## Abstract

In this study, composite nanofiber films comprising polyvinyl alcohol, wheat gluten, and glucose (PWG) were fabricated using electrospinning, followed by crosslinking via Maillard crosslinking. Various mass concentrations of ferulic acid (FA) were incorporated into PWG films. The results indicated that the average diameter of the FA-PWG films decreased from 449 nm to 331 nm as the concentration of FA increased, until reaching a concentration of 12%; after which, a significant increase in diameter was observed. The subsequent Fourier-transform infrared spectroscopy (FTIR), X-ray diffraction (XRD), and differential scanning calorimetry (DSC) results suggested that FA was distributed in the sample films in an amorphous form through hydrogen and ester bonds. Additionally, release experiments and antimicrobial tests on the FA-PWG sample films showed the good controlled release of FA and excellent anti-*Escherichia coli* and *Staphylococcus aureus* activity of this film. These findings all indicate that the FA-PWG nanofiber film is a potential candidate for active food packaging.

## 1. Introduction

In recent times, electrospun nanofiber films have gained widespread application in various fields, including bioscaffolds [1], drug delivery [2], and notably, active food packaging [3,4,5]. Active food packaging involves the integration of active molecules into packaging materials, leveraging the physicochemical properties of such molecules to safeguard foods [6]. The pivotal aspect of this process is the loading and subsequent release of the active molecules. Electrospinning offers distinct advantages, such as the ability to manipulate fiber morphology, achieve specific porosity, and attain a high volume-to-surface area ratio, rendering it an ideal technique for active packaging material preparation [7,8,9].

Materials used for food active packaging are often biodegradable polymers such as zein protein [10], chitosan [11], soy protein isolate [12], polyvinyl alcohol (PVA) [13], and wheat gluten (WG) [4,14,15]. Of these options, WG has garnered significant attention due to its exceptional water stability and unique viscoelasticity [16]. In addition, WG exhibits outstanding film-forming properties, and its molecular structure contains numerous inter/intra-chain disulfide and hydrogen bonds, rendering it a superior raw material for electrospinning [17]. PVA is frequently utilized as a fundamental component for electrospinning blends, owing to its favorable biocompatibility and full biodegradability [18]. Previous studies have proven that both are suitable raw materials for loading active molecules. For instance, Aziz et al. [19] developed a PVA/WG nanofiber film that was loaded with azathioprine, and the in vitro release outcomes indicated a reduction in the release of azathioprine following the initial sudden release, which was beneficial for the drug’s therapeutic efficacy.

Nevertheless, the deficiencies in thermal stability and mechanical properties of PVA/WG nanofiber films limit their further applications [15]. Therefore, we considered the use of the Maillard crosslinking reaction to overcome these deficiencies. It has been demonstrated that this reaction can enhance the mechanical property and thermal stability of sample films. Zhang et al. [4] prepared a WG/zein composite nanofiber film and found that the tensile strength was remarkably raised from 2.2 MPa to 9.5 MPa after xylose saccharification, and the denaturation temperature of the sample films was enhanced from 135.8 °C to 174.2 °C.

Ferulic acid (FA), also named 3-methoxy-4-hydroxycinnamic acid, is a polyphenol derivative that possesses antioxidant properties. This phenolic acid is widely distributed in fruits, wholegrains, and plant cell walls and is one of the effective constituents in several Chinese herbs, including Chuanxiong rhizome and Angelica sinensis [20]. It has obvious biological effects, such as antioxidant, free radical scavenging, anti-infection, and neuroprotection [13]. However, the poor water solubility and slow membrane absorption of FA significantly limit its utilization in the active food packaging field [21]. Interestingly, these deficiencies can be overridden by blending electrospinning with the appropriate polymer matrix.

Therefore, we loaded FA into PVA/WG/glucose (PWG) nanofiber films crosslinked by Maillard to achieve the sustainable release of FA while increasing the FA dissolution rate. The spinning solution was formulated by introducing varying concentrations of FA to the PWG mixed solution, followed by a comprehensive analysis of its physicochemical properties. The FA-PWG nanofiber films obtained after blending electrospinning were then heat-treated to achieve a Maillard crosslinking reaction. For the prepared sample films, a number of characterization analyses such as scanning electronic microscopy (SEM), Fourier-transform infrared (FTIR), X-ray diffraction (XRD), and differential scanning calorimetry (DSC) were performed. Then, the release characteristics of the FA-PWG films were studied using deionized water to simulate a high-moisture food matrix. Finally, typical food-borne pathogenic bacteria *Staphylococcus aureus* (*S. aureus*) and *Escherichia coli* (*E. coli*) were selected to verify the antimicrobial properties of the FA-PWG nanofiber films.

## 2. Materials and Methods

### 2.1. Materials

PVA (1788; Mw 19.8–26.4 kDa) with a degree of alcoholysis of 87.0–89.0% (mol/mol) was obtained from MREDA Technology Co., Ltd. (Beijing, China). WG with an Mw of 30–90 kDa (model TII) was provided by Tokyo Chemical Industry Co., Ltd. (Tokyo, Japan). Glucose (donated by Tianjin GreenBio Materials Co., Ltd., Tianjin, China) was used as a crosslinker for the Maillard reaction. *S. aureus* (CMCC26003) and *E. coli* (ATCC8739) were obtained from Beijing Sanyao Science & Technology and Nanjing Lezhen Biotechnology Co., Ltd. (Beijing, China).

### 2.2. Blending Electrospinning

A 27% (*w*/*v*) PVA/WG polymer was dissolved in a 50% acetic acid solution, with a mass ratio of 1:4. Then, glucose based on 20% of the mass of WG was added to the mixture to prepare a PWG solution. Subsequently, different masses of FA powders were added to the PWG solution to prepare FA-PWG mixed electrospinning solutions (FA levels: 0, 2, 4, 6, 8, 10, and 12 wt%, all weights based on WG), which were denoted FA0, FA2, FA4, FA6, FA8, FA10, and FA12, respectively.

All samples were loaded into medical syringes (5 mL) with 22G blunt needles and pumped through a syringe pump (Model HSP-101, MECC Co., Ltd., Fukuoka, Japan) with the flow rate of 1.0 mL/h. The electrospinning voltage (Spellman High Voltage, MECC Co., Ltd., Fukuoka, Japan) and collector to syringe tip distance were 25 kV and 10 cm. The FA-PWG nanofiber films obtained by electrospinning were collected on grounded square aluminum plates (15 × 15 cm) and then dried for 1 day at 25 °C. The entire process of electrospinning was controlled at 45% relative humidity and 25 °C. The resulting FA-PWG nanofiber films were thermally crosslinked at 150 °C for 3 h.

### 2.3. Physicochemical Properties of Electrospinning Solution

All samples were stirred overnight at 25 °C, and then, their surface tension, viscosity, and conductivity were measured. The conductivity was measured by a Malvern zeta potentiometer (ZS90, Malvern, Great Malvern, UK). The shear viscosity at a shear rate of 100 s^−1^ of electrospinning solutions was determined by a TA rheometer (Discovery HR10, TA Instruments, New Castle, DE, USA) with a parallel plate 50 mm in diameter. An interface tensiometer (Theta Flex, Biolin Scientific AB, Goteborg, Sweden) was applied to measure the surface tension of the FA-PWG nanofiber films.

### 2.4. Morphology

The microscopic surface morphology of each sample film was investigated by SEM (S-4800, HITACHI, Tokyo, Japan). Among them, the diameter distribution and average diameter of the sample film was measured by the measurement of 50 fibers in 3 randomly elected SEM pictures using ImageJ software.

### 2.5. Compatibility of Components

The infrared spectra of all sample films were measured with a Nicolet IS10 instrument (Thermo Fisher Scientific, Waltham, MA, USA). The wavenumber range, resolution, and scan times of FTIR were 400–4000 cm^−1^, 4 cm^−1^, and 32, respectively.

### 2.6. Physical Form

The XRD curve of the sample film was determined by a D8 Advance diffractometer (Bruker AXS, Karlsruhe, Germany). The operating inclination, tube current, and tube voltage of the CuK α radiation source were 0.02°, 40 mA, and 40 kV, respectively. The measured wide angle diffraction range was 5° to 60° (2θ).

The DSC curves of the sample films were measured using DSC8000 equipment (PerkinElmer, Waltham, MA, USA). The sample film (about 5 mg) was sealed in an aluminum crucible (40 μL). The films were heated from 30 °C to 300 °C with a nitrogen atmosphere (0.05 L/min) at a heating rate of 10 °C/min.

The mechanical properties of the FA-PWG nanofiber films were determined by an AGS-J universal testing machine (Shimadzu, Fukuoka, Japan). The sample films were cut into 3 cm × 1 cm strips, and the thickness of the films was controlled to be around 0.15 mm. The whole testing process was carried out at room temperature, and the rate of controlled stretching was 5 mm/min.

### 2.7. Active Molecules Release

The release behavior of the FA-PWG nanofiber films in deionized water over 1440 min was investigated by modifying the methods reported by Huang et al. [21]. The FA-PWG nanofiber film was weighed as 25 mg (±0.1 mg) each, placed in a beaker containing ultrapure water (50 mL), and stirred at 37 °C in a water bath with a magnetic stirrer (100 rpm). At specified time intervals (every 10 min for the first 60 min and every 60 min thereafter), the supernatant (5 mL) was removed from the beaker, and fresh ultrapure water (5 mL) was added. The absorbance value of the supernatant was measured by a UV–Vis spectrophotometer (Agilent Technologies Cary 100, Santa Clara, CA, USA) at 321 nm. The measured absorbances were brought to the predetermined standard curve: Y = 0.0061X + 0.011 (R^2^ = 0.9986), where the X value was the concentration of FA (μg/mL), and the Y value was the absorbance at 321 nm. The cumulative release of FA at that moment was calculated according to Equation (1):

(1) $$\mathrm{Accumulative}\;\mathrm{release}\;\left(\%\right)=\frac{W_{t}}{W_{d}}\times 100\%$$

where W_t_ is the release amount of FA in time t, and W_d_ is the whole loading amount of FA in the nanofiber film.

The spreading, solubilizing, and eroding mechanisms of the active molecules are the most essential rate governing mechanisms for release control products [22]. For better representation of the release mechanism of FA from the PWG sample films, four release kinetic models, including First-order (Equation (2)), Zero-order (Equation (3)), Korsmeyer–Peppas (Equation (4)), and Higuchi (Equation (5)), were used to study the FA-PWG nanofiber films [23,24,25,26].

(2) $$\ln(1-M_{t}/M_{\infty })=-k_{F}t$$

(3) $$M_{t}/M_{\infty }=k_{Z}t$$

(4) $$M_{t}/M_{\infty }=k_{K}t^{n}$$

(5) $$M_{t}/M_{\infty }=k_{H}t^{0.5}$$

M_t_/M_∞_ are the release ratios of the FA molecules at moments t; K_F_, K_Z_, K_K_, and K_H_ are the constant parameters of the First-order, Zero-order, Korsmeyer–Peppas, and Higuchi equations, respectively; and n is the diffusion index.

### 2.8. Antimicrobial Test

The plate agar disc diffusion methods were applied to evaluate the antimicrobial performance of the FA-PWG sample films. After culturing *S. aureus* and *E. coli* to fourth-generation strains, gradient dilutions (~10^6^ CFU/mL) were performed. A dilution (about 100 μL) was taken for the plate coating, and a triangular glass rod was used to disperse the bacterial solution evenly. The sample film electrospun on aluminum foil was cut into 6 mm circular discs and disinfected using UV for 1 h. Then, the FA-PWG nanofiber films were placed on a coated plate and incubated at 37 °C for 24 h in a constant temperature incubator. The diameter of the inhibition zone was measured on the X and Y planes using ImageJ V 1.8.0 software. Three plate samples were coated at each FA concentration.

The relative concentrations of *E. coli* and *S. aureus* correlated with the absorbance of the bacterial suspension at 600 nm over a range of concentrations. The UV–Vis spectrophotometer (Agilent Technologies Cary 100, Santa Clara, CA, USA) was used to determine the absorbance at 600 nm of the bacterial suspension (~10^6^ CFU/mL), which was recorded as the relative concentration of 1.0. Then, the absorbance at 600 nm was measured at relative concentrations of 0.1, 0.2, 0.4, and 0.8, respectively. The equations of the standard curves for *E. coli* and *S. aureus* were obtained as Y_1_ = 1.412X_1_ + 0.158 (R^2^ = 0.998, where X_1_ is the relative concentration, and Y_1_ is the absorbance of *E. coli* suspension at 600 nm) and Y_2_ = 1.652X_2_ + 0.0214 (R^2^ = 0.998, where X_2_ is the relative concentration, and Y_2_ is the absorbance of *S. aureus* suspension at 600 nm), respectively. Take 100 μL of the bacterial suspension, dilute 100 times, and add the sample film (20 mg) that has been pretreated by UV irradiation and place it in the shaker (200 rpm, 37 °C) to activate for 24 h. Finally, the absorbance of each activation solution at 600 nm was measured and brought into the standard curve equation to obtain the relative concentration.

### 2.9. Statistical Analysis

Statistical analysis was performed by IMB SPSS Statistics 26 software, and the results were reported as the mean ± standard deviation (SD). One-way analysis of variance (ANOVA) and Duncan analysis were used for data analysis. Significant difference was considered at *p* < 0.05. All experiments were performed in triplicate. The number of samples per experimental replicate was three.

## 3. Results and Discussion

### 3.1. Solution Properties

The success of electrospinning is contingent upon the physicochemical property of the electrospinning solution, specifically its surface tension, viscosity, and conductivity [7]. Table 1 shows the physicochemical properties of the PWG electrospinning solutions with different concentrations of FA. It was not difficult to see that, with the augmentation of the FA mass concentration in the mixed solvent, the surface tension and viscosity of the FA-PWG spinning solution was boosted. The surface tension of the spinning solution often affects the final morphology of nanofibers by influencing the Taylor cone formation during the electrospinning process. In general, an increase in solution surface tension is not conducive to the electrospinning process [16]. Additionally, the conductivity reaches the maximum at FA10, which is 1.28 ms/cm. There were no significant differences in the physicochemical properties between the FA0 and FA2 electrospinning solutions (*p* > 0.05). With the increasing addition (from FA0 to FA12), the viscosity increased continuously from 2.01 to 2.45 Pa.s. The increase in solution viscosity can be explained by the formation of molecular clusters between PVA/WG/G-FA, which increases the degree of entanglement between polymer molecular chains in a solution, thereby enhancing the electrospinning ability of the solution [27]. However, the conductivity in the FA-PWG solution showed a downward trend in the FA12 solution.

Excessive solution viscosity or inadequate conductivity will hinder the stretching of the fiber during electrospinning, making the resulting nanofibers thicker [16]. Within a specific range, higher solution conductivity facilitates greater fiber stretching and differentiation, resulting in smaller fiber diameters. Niloufar et al. [28] prepared FA-cyclodextrin inclusion complex nanofiber pad and found that the conductivity of the sample increased at first and then decreased with the increasing FA concentration, which was consistent with the results of this experiment.

### 3.2. Morphology

**Figure 1:**
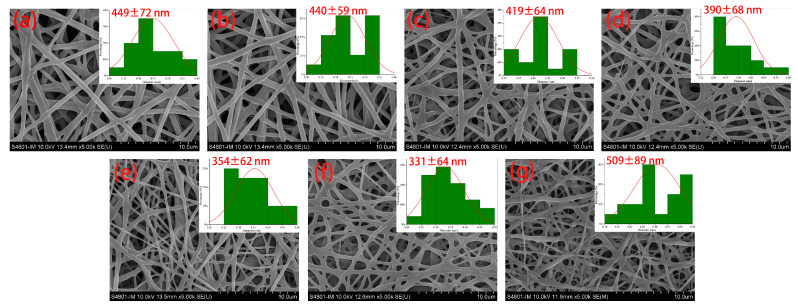
SEM images of PVA/WG/G composite nanofiber films loaded with different concentrations of FA: (**a**) FA0; (**b**) FA2; (**c**) FA4; (**d**) FA6; (**e**) FA8; (**f**) FA10; (**g**) FA12. Data are presented as the mean values ± standard deviation.

An analysis of the SEM images reveals that the average diameter of FA-PWG nanofiber films exhibits a trend of initially decreasing and subsequently increasing with an increase in the FA loading concentration (Figure 1). Notably, the FA-PWG nanofiber films exhibit relatively smooth surfaces, and the absence of granular substances on the fiber surface suggests that FA has been uniformly dispersed in the PWG matrix. The average diameter of nanofiber film FA0 is 449 nm; when the addition of FA is 10%, the diameter of nanofiber film FA10 is the smallest, which is 331 nm. However, when the addition of FA reaches 12%, the average diameter of the nanofiber film FA12 increases to 509 nm. The difference in average diameter distribution can be attributed to the different physicochemical properties of each electrospinning solution due to the addition of different FA concentrations. For example, the average diameter of the FA10 nanofiber film is the smallest, and the diameter distribution is relatively uniform. This can be attributed to the high conductivity of the electrospinning solution at this time, which is conducive to the full stretching of the fibers during the electrospinning process. These findings align with Asli et al.’s research, which similarly found that pure hydroxypropyl-γ-cyclodextrin nanofibers had a greater average diameter than the FA/cyclodextrin inclusion complex [29]. This disparity was owed to the higher conductivity and lower solution viscosity than the hydroxypropyl-γ-cyclodextrin solution of the FA/cyclodextrin inclusion complex solution. As we all know, a decrease in the fiber diameter is not only conducive to the full crosslinking reaction, so that the non-protonated amino and electrophilic carbonyl groups can be fully exposed, but also improves the loading of nanofiber films to active molecules [30]. Deng et al. [31] prepared gelatin/zein nanofiber films loaded with allopurinol and observed that the composite nanofibers loaded with 2.5% allopurinol had the smallest average diameter and the highest entrapment efficiency, which were 244.1 nm and 94.92%.

### 3.3. Component Interaction Analysis

**Figure 2:**
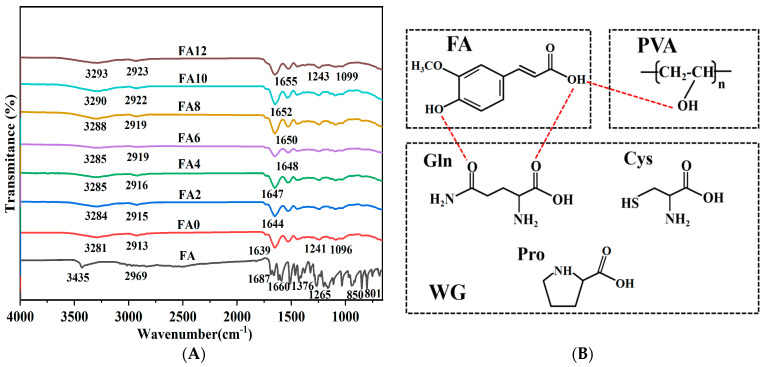
(**A**) FTIR spectra of FA powder and PVA/WG/G composite nanofiber films loaded with different concentrations of FA. (**B**) The molecular formulas of FA, PVA, and WG.

The FTIR spectra of the FA and FA-PWG nanofiber films are presented in Figure 2A. The characteristic peaks of FA at 1687 cm^−1^ and 1660 cm^−1^ are formed by the stretching vibration of C=O, which indicates the presence of carbonyl groups in the crystal lattice of FA [21]. The characteristic absorption peak at 3435 cm^−1^ is related to the O-H stretching vibration. Additionally, the characteristic absorption peaks of FA at 2969 cm^−1^ and 1265 cm^−1^ are related to the C-H stretching vibration and the C-O-C asymmetric stretching vibration [32]. The characteristic absorption peak at 1376 cm^−1^ is related to the aromatic nucleus in the FA molecule, and the characteristic absorption peaks at 850 cm^−1^ and 801 cm^−1^ are caused by two adjacent H atoms on the benzene ring of the FA molecule [33]. These chemical bonds in different vibrational forms reflect the ordered crystal structure of FA molecules. However, for the nanofiber film FA-PWG, the characteristic peak of FA in the infrared spectrum disappears, indicating that FA in the nanofiber film does not form the FA dimer necessary for lattice construction [21,34].

Firstly, with the increasing FA addition, the O-H stretching vibration peak in the FA-PWG nanofiber films shifted from 3281 (FA0) to 3285 (FA6) and 3290 cm^−1^ (FA10). Secondly, the stretching vibration peak of the WG amide I band in the FA-PWG nanofiber films shifted from 1639 (FA0) to 1644 (FA2) and 1655 cm^−1^ (FA12) higher wavenumbers. These phenomena are due to the chemical interaction between FA and the components of the FA-PWG film, and it is speculated that this interaction is a hydrogen bond (Figure 2B) [13,29,35]. Finally, the addition of FA also leads to a weakening of the N-H bending vibrations associated with the amide II band, which may be related to the presence of esterification reactions between some of the amino acids in WG (e.g., Ser and Tyr) and FA [3].

### 3.4. Physical State Analysis

**Figure 3:**
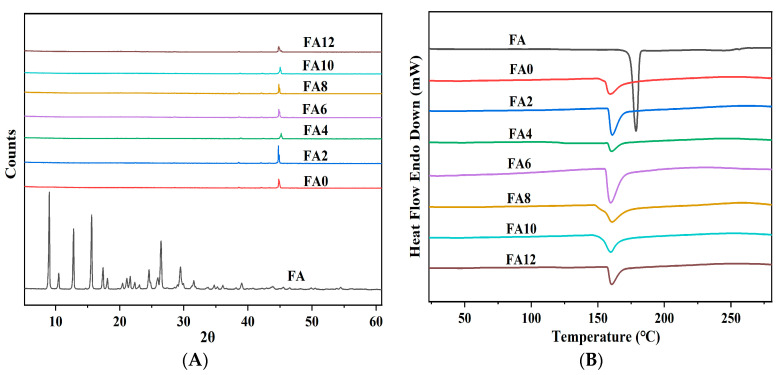
(**A**) XRD patterns and (**B**) DSC thermograms of FA powder and PVA/WG/G composite nanofiber films loaded with different concentrations of FA.

The utilization of X-ray diffraction (XRD) analysis is a common practice in the characterization of the crystalline state of a substance. The amorphous dispersion of active molecules in nanofibers is generally regarded as advantageous for their dissolution and release [36]. Depicted in Figure 3A are the XRD images of the FA and FA-PWG nanofiber films. It can be clearly observed that FA has many diffraction peaks of different intensities, indicating that it is a crystalline substance [37]. The diffraction pattern of the FA-PWG nanofiber films exhibits a resemblance to FA0. The absence of a characteristic diffraction peak associated with the crystalline phase of FA suggests the existence of amorphous FA in the FA-PWG nanofiber film. This may be connected with the chemical interaction between FA and WG during the process of electrospinning. It is widely acknowledged that the quick evaporation of a solvent during electrospinning hinders the crystallization of active molecules [38]. In contrast, the FA/hydroxypropyl-β-cyclodextrin composite nanofiber films prepared by Asli et al. [29] still contained some noncomplex FA crystals. The amorphous distribution of FA in the composite nanofiber films is consistent with the notion of loading active molecules into functional fiber food packaging and the controllable release characteristics of active molecules [21]. Therefore, the FA-PWG nanofiber films prepared in this study are suitable for active packaging materials.

Figure 3B shows the DSC results of the FA and FA-PWG nanofiber films, and the results are in full agreement with those of XRD. FA has a sharp thermal absorption peak at 174.38 °C, indicating its crystal state. Nonetheless, the FA-PWG nanofiber films did not exhibit a thermal absorption peak of FA, which indicates that the active molecule was present in an amorphous state within the fibers. The melting temperatures of the FA-PWG nanofiber films loaded with different concentrations of FA were all around 160.49 °C, which was consistent with that of FA0. It was indicated that the thermodynamic stability of the composite nanofiber films was not affected after loading FA. The shape of the DSC curve of the FA-PWG nanofiber films loaded with FA, although consistent with that of FA0, corresponded to a different value of enthalpy change. This may be related to the chemical interaction (hydrogen bond formation) between active molecule FA and the components of the composite nanofiber films [39].

**Figure 4:**
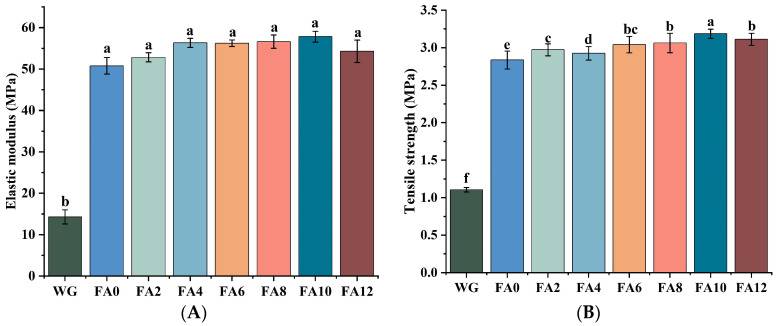
(**A**) Elastic modulus and (**B**) tensile strength of WG alone and PVA/WG/G composite nanofiber films loaded with different concentrations of FA. Different lowercase letters in the same chart indicate significant differences (*p* < 0.05).

Figure 4A,B show the elastic modulus and tensile strength of WG alone electrospinning and FA-PWG nanofiber films loaded with different concentrations of FA. The elastic modulus and tensile strength of the nanofiber films electrospun by WG alone are low, and the mechanical strength is poor. However, the elastic modulus and tensile strength of the FA-PWG nanofiber films were greatly improved. This could be attributed to the addition of the co-spun polymer PVA and the crosslinker glucose. There was no significant difference in the elastic modulus among FA0–FA12, and the tensile strength of FA10 was the highest, which was 3.186 MPa. This could be attributed to the fact that the average diameter of the FA10 nanofiber film was the smallest, which led to a higher degree of crosslinking between the fibers and the formation of a denser and stiffer network structure. This was consistent with the findings of Zhang et al. [4].

### 3.5. Release Performance

**Figure 5:**
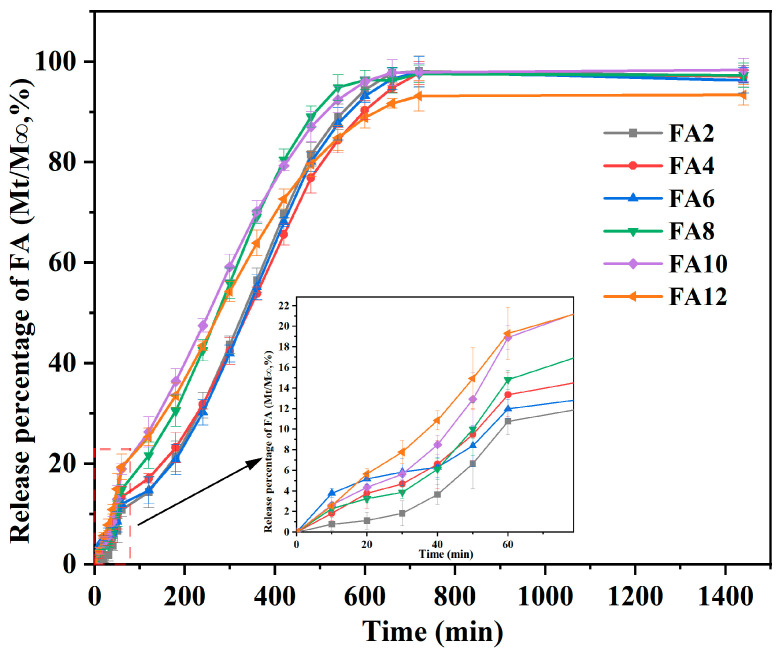
Release curves of the FA-PWG composite nanofiber films loaded with different concentrations of FA.

The release curve of the FA-PWG nanofiber film is presented in Figure 5. In the initial 60 min, each FA-PWG nanofiber film showed a rapid release behavior, with a release rate of about 14%, which might be related to the dissolution of FA adhered to the fiber surface or embedded in a shallow position inside the fiber. In another study, a PVA nanofiber film loaded with γ-cyclodextrin/FA inclusion complex prepared by Vimalasruthi et al. [13] did not have a fast release behavior, and the release rate of FA was less than 6% at 60 min. During 60–720 min, the release percentage of FA in the composite nanofibers increased slowly over time. After 720 min, the FA release rate tended to be stable, and the release percentage of FA10 was the highest, which was 97.38%. The aforementioned occurrence may be ascribed to the continuous relaxation and disintegration of the interior of the composite nanofibers under the attack of water molecules over time, thereby slowly exposing the FA located inside the fibers. Gyuldzhan et al. [40] prepared polycaprolactone/chitosan composite nanofiber films loaded with FA by electrospinning. Their in vitro release findings indicated that the highest rate of FA release was merely 91.4%, which was obviously lower than the rate observed in the current study.

For FA12, it has the lowest final release rate of only 93.24%, which may be related to the diameter distribution of the composite nanofibers. The relationship between fiber diameter and specific surface area is widely acknowledged, as smaller diameters result in increased surface contact between active molecules and dissolution medium solutions, leading to higher release rates [41]. These FA-PWG nanofiber films with both an initial blast release and later sustained release properties exhibit significant possibilities for employment in active food packaging. They are ideally suited for food packaging that is prone to rapid oxidation (peeled fruits, e.g., apples, bananas, etc.), as well as for packaging that requires sustained and effective antioxidant properties (meat and vegetables, e.g., cherry tomatoes, steaks, etc.) [21]. WG may pose a food safety risk to certain populations susceptible to celiac disease, which is mainly related to the thirty-one and nine amino acid peptide sequences of the gliadin in WG [42]. However, it has been shown that the process of electrospinning is expected to reduce this risk as far as the mechanism of electrospinning is concerned [16].

**Figure 6:**
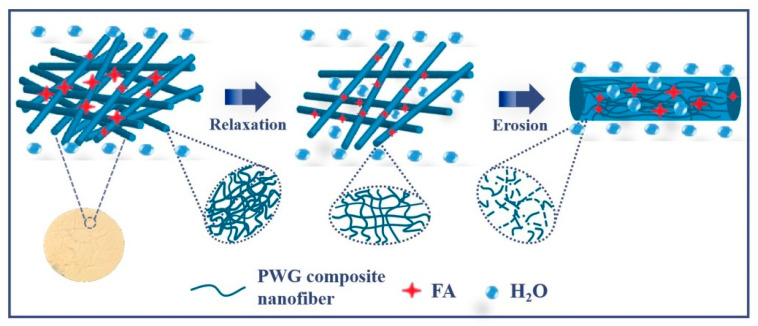
Schematic diagram of the release characterization of active molecule FA.

As shown in Table 2, all model release kinetic parameters of the samples were compared, and the Korsmeyer–Peppas model had a high coefficient of determination (R^2^ > 0.95), suggesting that this model was more suitable for describing the release of FA in PWG composite nanofibers. In the Korsmeyer–Peppas model, when n ≤ 0.45, the corresponding release mechanism is the Fickian diffusion mechanism [43]. If the value of n falls between 0.45 and 0.89, the mechanism is non-Fickian, where the diffusion mechanism is mainly controlled by a combination of the relaxation and diffusion of macromolecular chain segments [44]. When n = 0.89, the kinetics is the Zero-order release model, which is ideal, implying the complete release of the encapsulated active molecules. When n > 0.89, the release mechanism is the super Case II release mechanism [45]. At this time, the release of the active molecule FA follows complex release mechanisms, including diffusion, expansion, and erosion, in which the erosion of the carrier is mainly dominant. Thus, the release of FA-PWG nanofiber films in ultrapure water is a complex process, in which the crosslinked relaxation and erosion of the negative carrier nanofiber films dominate (Figure 6).

### 3.6. Antibacterial Property Analysis

**Figure 7:**
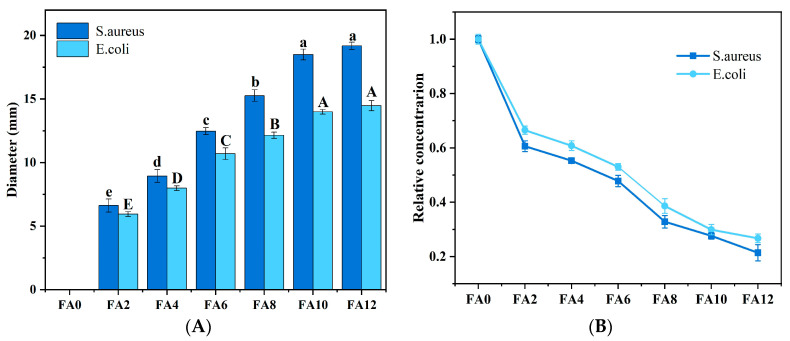
(**A**) Diameter of the inhibition zones of the sample films. Different letters in the same set of charts indicate significant differences (*p* < 0.05). (**B**) Bactericidal effects of the sample films in a liquid medium.

Figure 7A presents the diameter of the inhibitory zone of the FA-PWG nanofiber films. It can be clearly seen that FA0 has no inhibition function on both *S. aureus* and *E. coli*, and the sample film with a FA addition has a better inhibition function on *S. aureus* than *E. coli*. The same phenomenon also appeared in the research of Jia et al. [32]. With the increase in FA loading in the sample films, the diameter of the inhibitory circle of the FA-PWG nanofiber film enhanced continuously. At FA12, the diameter of the inhibitory zone of the sample film reached the maximum of 1.918 cm for *S. aureus* and 1.447 cm for *E. coli*. In another study, the maximum zone of the inhibition of *S. aureus* was only 1.65 cm in diameter for the FA/hyaluronan/polyvinylpyrrolidone composite nanofiber film prepared by Grimaudo et al. [46]. It was not difficult to find that the inhibition effect increased significantly (*p* < 0.05) at 2% to 10% of the FA addition, while no obvious differences were observed when the FA level was 12%. The observed phenomenon can be ascribed to the augmentation in the diameter of the FA12 nanofiber film, which hinders the diffusion of active molecules, and the diffusion resistance of the agar medium [15]. The results were similar to the bactericidal effect of the film in a liquid medium. Figure 7B shows the relative concentrations of the FA-PWG nanofiber films loaded with different FA concentrations in a liquid medium after 24 h of activation. The results showed that, as the FA loading of the nanofiber films increased, the bactericidal effect of the films in the liquid culture medium also improved remarkably. The relative concentrations of the *S. aureus* and *E. coli* suspensions after 24 h of activation were only 0.214 and 0.267 in the liquid medium added to sample film FA12. Currently, the bactericidal mechanism of FA is generally believed to be through the modification of bacterial cell membranes, changing the hydrophobicity of cell membranes and resulting in the localized rupture of bacterial cell membranes, thus providing the bactericidal effects [32,40].

## 4. Conclusions

In this study, PWG nanofiber films loaded with active molecular FA were prepared by blended electrospinning. In the group of FA0–FA10, the diameter of the sample nanofiber film decreased, continuously along with an increase in the solution conductivity. However, for FA12, the increase in solution viscosity and the decrease in conductivity led to an increase in the nanofiber film diameter. The findings from the FTIR, XRD, and DSC analyses suggested that chemical interactions (e.g., esterification reactions and hydrogen bonding) existed between FA and various components of the FA-PWG film and were distributed in the film in an amorphous form. Subsequent release studies revealed that the Korsmeyer–Peppas model accurately revealed the release of FA in the PWG sample film, and the release process mostly consisted of carrier swelling and erosion. Antibacterial tests showed that the sample films had excellent antibacterial properties and were better resistant to *S. aureus* than *E. coli.* Therefore, FA-PWG nanofiber films can be candidates for antimicrobial food packaging materials, and they are most suitable for foods with a high water content.

## Figures and Tables

**Table 1:** Characterization of the physicochemical properties of the PVA/WG/G electrospinning solution with different concentrations of FA.

Sample (FA-PWG)	Conductivity (ms/cm)	Viscosity (Pa.s)	Surface Tension (mN/m)
FA0	1.03 ± 0.05 ^e^	2.01 ± 0.08 ^e^	29.81 ± 0.13 ^d^
FA2	1.06 ± 0.04 ^de^	2.06 ± 0.02 ^e^	29.92 ± 0.48 ^cd^
FA4	1.15 ± 0.02 ^bc^	2.15 ± 0.05 ^d^	30.96 ± 0.71 ^cd^
FA6	1.18 ± 0.02 ^bc^	2.18 ± 0.02 ^cd^	31.26 ± 0.95 ^bcd^
FA8	1.23 ± 0.04 ^ab^	2.26 ± 0.03 ^c^	31.57 ± 0.28 ^bc^
FA10	1.28 ± 0.02 ^a^	2.36 ± 0.04 ^b^	32.48 ± 0.47 ^b^
FA12	1.12 ± 0.07 ^cd^	2.45 ± 0.05 ^a^	34.19 ± 0.96 ^a^

All values are the mean ± standard deviation of three replicates. Different lowercase letters in the same column indicate significant differences (*p* < 0.05). FA: ferulic acid; PVA: polyvinyl alcohol; WG: wheat gluten; G: glucose.

**Table 2:** Kinetic parameters of FA release from the prepared composite nanofiber films.

Formulation	Zero-Order		First-Order		Higuchi		Korsmeyer–Peppas			Release Rate (%)
	K_Z_	R^2^	K_F_	R^2^	K_H_	R^2^	K_K_	n	R^2^	
FA2	0.0904	0.6931	0.0034	0.7578	4.0457	0.8533	0.0501	1.1809	0.9844	97.15
FA4	0.0901	0.7499	0.0031	0.7962	3.7053	0.8884	0.3171	0.8696	0.9796	96.25
FA6	0.0906	0.7314	0.0032	0.7721	3.7247	0.8661	0.3823	0.8393	0.9664	96.17
FA8	0.0902	0.6986	0.0033	0.7623	3.8357	0.8853	0.2083	0.9697	0.9765	96.37
FA10	0.1186	0.5302	0.0034	0.8167	3.7368	0.9007	0.3263	0.9018	0.9686	97.38
FA12	0.0815	0.732	0.0025	0.8219	3.4398	0.9141	0.7767	0.7303	0.9737	93.24

All values are the means of three replicates. K_Z_, K_F_, K_H_, and K_K_ are the constant parameters of the Zero-order, First-order, Higuchi, and Korsmeyer–Peppas equations, respectively; R^2^ is the coefficient of determination; and n is diffusion index. FA: ferulic acid.

## Data Availability

All data are contained within the article.

## References

[1] Wen P., Wen Y., Zong M.H., Linhardt R.J., Wu H. (2017). Encapsulation of Bioactive Compound in Electrospun Fibers and Its Potential Application. J. Agric. Food Chem..

[2] Kumar C.S., Soloman A.M., Thangam R., Perumal R.K., Gopinath A., Madhan B. (2020). Ferulic acid-loaded collagen hydrolysate and polycaprolactone nanofibres for tissue engineering applications. Iet Nanobiotechnol..

[3] Li T., Xia N., Xu L.N., Zhang H., Zhang H.J., Chi Y.J., Zhang Y.L., Li L.L., Li H.Y. (2021). Preparation, characterization and application of SPI-based blend film with antioxidant activity. Food Packag. Shelf Life.

[4] Zhang Y., Deng L., Zhong H., Zou Y.C., Qin Z.Y., Li Y., Zhang H. (2022). Impact of glycation on physical properties of composite gluten/zein nanofibrous films fabricated by blending electrospinning. Food Chem..

[5] Wang P., Li Y., Zhang C., Que F., Weiss J., Zhang H. (2020). Characterization and antioxidant activity of trilayer gelatin/dextran-propyl gallate/gelatin films: Electrospinning versus solvent casting. LWT-Food Sci. Technol..

[6] Li W.H., Zhang C., Chi H., Li L., Lan T.Q., Han P., Chen H.Y., Qin Y.Y. (2017). Development of Antimicrobial Packaging Film Made from Poly(Lactic Acid) Incorporating Titanium Dioxide and Silver Nanoparticles. Molecules.

[7] Haider A., Haider S., Kang I.K. (2015). A comprehensive review summarizing the effect of electrospinning parameters and potential applications of nanofibers in biomedical and biotechnology. Arab. J. Chem..

[8] Xue J., Wu T., Dai Y., Xia Y. (2019). Electrospinning and Electrospun Nanofibers: Methods, Materials, and Applications. Chem. Rev..

[9] Cen Z., Yang L., Peng W., Hui Z. (2020). Electrospinning of nanofibers: Potentials and perspectives for active food packaging. Compr. Rev. Food Sci. Food Saf..

[10] Liu F.G., Li X.Z., Wang L., Yan X.J., Ma D.X., Liu Z.G., Liu X.B. (2020). Sesamol incorporated cellulose acetate-zein composite nanofiber membrane: An efficient strategy to accelerate diabetic wound healing. Int. J. Biol. Macromol..

[11] Riaz A., Lagnika C., Luo H., Dai Z.Q., Nie M.M., Hashim M.M., Liu C.Q., Song J.F., Li D.J. (2020). Chitosan-based biodegradable active food packaging film containing Chinese chive (*Allium tuberosum*) root extract for food application. Int. J. Biol. Macromol..

[12] Nassar S.F., Dombre C., Gastaldi E., Touchaleaume F., Chalier P. (2018). Soy protein isolate nanocomposite film enriched with eugenol, an antimicrobial agent: Interactions and properties. J. Appl. Polym. Sci..

[13] Vn A., Mkm B., St A. (2020). Electrospinning preparation and spectral characterizations of the inclusion complex of ferulic acid and γ-cyclodextrin with encapsulation into polyvinyl alcohol electrospun nanofibers. J. Mol. Struct..

[14] Zhang Y., Deng L., Zhong H., Pan J., Li Y., Zhang H. (2020). Superior water stability and antimicrobial activity of electrospun gluten nanofibrous films incorporated with glycerol monolaurate. Food Hydrocoll..

[15] Wang H., She Y., Chu C., Liu H., Jiang S., Sun M., Jiang S. (2015). Preparation, antimicrobial and release behaviors of nisin-poly (vinyl alcohol)/wheat gluten/ZrO_2_ nanofibrous membranes. J. Mater. Sci..

[16] Zhang H.J., Jin C.M., Lv S.H., Ren F.Y., Wang J. (2023). Study on electrospinning of wheat gluten: A review. Food Res. Int..

[17] Han Y., Chen H. (2013). Enhancement of nanofiber elasticity by using wheat glutenin as an addition. Polym. Sci..

[18] Ugur M.H., Oktay B., Gungor A., Kayaman-Apohan N. (2018). Highly thermally resistant, hydrophobic poly(vinyl alcohol)-silica hybrid nanofibers. J. Serbian Chem. Soc..

[19] Aziz S., Hosseinzadeh L., Arkan E., Azandaryani A.H. (2019). Preparation of electrospun nanofibers based on wheat gluten containing azathioprine for biomedical application. Int. J. Polym. Mater..

[20] Zhao Z.H., Moghadasian M.H. (2008). Chemistry, natural sources, dietary intake and pharmacokinetic properties of ferulic acid: A review. Food Chem..

[21] Huang X.Y., Jiang W.L., Zhou J.F., Yu D.G., Liu H. (2022). The Applications of Ferulic-Acid-Loaded Fibrous Films for Fruit Preservation. Polymers.

[22] Langer R., Peppas N. (1983). Chemical and Physical Structure of Polymers as Carriers for Controlled Release of Bioactive Agents: A Review. Polym. Rev..

[23] Lobo C. (2001). Modeling and comparison of dissolution profiles. Eur. J. Pharm. Sci..

[24] Neo Y.P., Swift S., Ray S., Gizdavic-Nikolaidis M., Jin J.Y., Perera C.O. (2013). Evaluation of gallic acid loaded zein sub-micron electrospun fibre mats as novel active packaging materials. Food Chem..

[25] Higuchi T. (1963). Theoretical analysis of rate of release of solid drugs dispersed in solid matrices. J. Pharm. Sci..

[26] Korsmeyer R.W., Gurny R., Doelker E., Buri P., Peppas N.A. (1983). Mechanisms of Solute Release from Porous Hydrophilic Polymers. Int. J. Pharm..

[27] Yang J.L., Yu K., Tsuji T., Jha R., Zuo Y.Y. (2019). Determining the surface dilational rheology of surfactant and protein films with a droplet waveform generator. J. Colloid Interface Sci..

[28] Sharif N., Golmakani M.T., Niakousari M., Hosseini S.M.H., Ghorani B., Lopez-Rubio A. (2018). Active Food Packaging Coatings Based on Hybrid Electrospun Gliadin Nanofibers Containing Ferulic Acid/Hydroxypropyl-Beta-Cyclodextrin Inclusion Complexes. Nanomaterials.

[29] Celebioglu A., Uyar T. (2020). Development of ferulic acid/cyclodextrin inclusion complex nanofibers for fast -dissolving drug delivery system. Int. J. Pharm..

[30] Irani M., Sadeghi G.M.M., Haririan I. (2017). The sustained delivery of temozolomide from electrospun PCL-Diol-b-PU/gold nanocompsite nanofibers to treat glioblastoma tumors. Mater. Sci. Eng. C-Mater. Biol. Appl..

[31] Deng L.L., Li Y., Feng F.Q., Wu D., Zhang H. (2019). Encapsulation of allopurinol by glucose cross-linked gelatin/zein nanofibers: Characterization and release behavior. Food Hydrocoll..

[32] Jia Q.Q., Lin X.H., Yang Y.W., Duan B. (2023). Multifunctional edible chitin nanofibers/ferulic acid composite coating for fruit preservation. J. Polym. Sci..

[33] Panwar R., Sharma A.K., Kaloti M., Dutt D., Pruthi V. (2016). Characterization and anticancer potential of ferulic acid-loaded chitosan nanoparticles against ME-180 human cervical cancer cell lines. Appl. Nanosci..

[34] Yang H., Feng K., Wen P., Zong M.H., Lou W.Y., Wu H. (2017). Enhancing oxidative stability of encapsulated fish oil by incorporation of ferulic acid into electrospun zein mat. Lwt-Food Sci. Technol..

[35] Narayanan G., Boy R., Gupta B.S., Tonelli A.E. (2017). Analytical techniques for characterizing cyclodextrins and their inclusion complexes with large and small molecular weight guest molecules. Polym. Test..

[36] Yu D.G., Li J.J., Williams G.R., Zhao M. (2018). Electrospun amorphous solid dispersions of poorly water-soluble drugs: A review. J. Control. Release.

[37] Huang W.D., Yang Y.Y., Zhao B.W., Liang G.Q., Liu S.W., Liu X.L., Yu D.G. (2018). Fast Dissolving of Ferulic Acid via Electrospun Ternary Amorphous Composites Produced by a Coaxial Process. Pharmaceutics.

[38] Kumar N., Goel N. (2019). Phenolic acids: Natural versatile molecules with promising therapeutic applications. Biotechnol. Rep..

[39] Kim Y.J., Park M.R., Kim M.S., Kwon O.H. (2012). Polyphenol-loaded polycaprolactone nanofibers for effective growth inhibition of human cancer cells. Mater. Chem. Phys..

[40] Yakub G., Ignatova M., Manolova N., Rashkov I., Markova N. (2018). Chitosan/ferulic acid-coated poly(ε-caprolactone) electrospun materials with antioxidant, antibacterial and antitumor properties. Int. J. Biol. Macromol..

[41] Yu D.G., Yang J.M., Branford-White C., Lu P., Zhang L., Zhu L.M. (2010). Third generation solid dispersions of ferulic acid in electrospun composite nanofibers. Int. J. Pharm..

[42] Shewry P.R., Tatham A.S. (2016). Improving wheat to remove coeliac epitopes but retain functionality. J. Cereal Sci..

[43] Kajdic S., Planinsek O., Gasperlin M., Kocbek P. (2019). Electrospun nanofibers for customized drug-delivery systems. J. Drug Deliv. Sci. Technol..

[44] Kalu V.D., Odeniyi M.A., Jaiyeoba K.T. (2007). Matrix properties of a new plant gum in controlled drug delivery. Arch. Pharmacal Res..

[45] Ferrari P.C., Oliveira G.F., Chibebe F.C.S., Evangelista R.C. (2009). In vitro characterization of coevaporates containing chitosan for colonic drug delivery. Carbohydr. Polym..

[46] Grimaudo M.A., Concheiro A., Alvarez-Lorenzo C. (2020). Crosslinked Hyaluronan Electrospun Nanofibers for Ferulic Acid Ocular Delivery. Pharmaceutics.

